# Gut Microbiota and Short-Chain Fatty Acid Profile between Normal and Moderate Malnutrition Children in Yogyakarta, Indonesia

**DOI:** 10.3390/microorganisms9010127

**Published:** 2021-01-07

**Authors:** Rafli Zulfa Kamil, Agnes Murdiati, Mohammad Juffrie, Jiro Nakayama, Endang Sutriswati Rahayu

**Affiliations:** 1 Department of Food and Agricultural Product Technology, Faculty of Agricultural Technology, Universitas Gadjah Mada, Jl. Flora No 1 Bulaksumur, Yogyakarta 55281, Indonesia; rafli.zulfa.k@mail.ugm.ac.id (R.Z.K.); amurdiati@ugm.ac.id (A.M.); 2 Center for Food and Nutrition Studies, Universitas Gadjah Mada, Jl. Teknika Utara Barek, Yogyakarta 55281, Indonesia; 3 Center of Excellence for Probiotics, Universitas Gadjah Mada, Jl. Teknika Utara Barek, Yogyakarta 55281, Indonesia; 4 Faculty of Medicine, Public Health and Nursing, Universitas Gadjah Mada, Jl. Farmako, Senolowo, Sekip Utara, Yogyakarta 55281, Indonesia; mjuffrie@ugm.ac.id; 5 Department of Bioscience and Biotechnology, Faculty of Agriculture, Kyushu University, 6-10-1, Hakozaki, Higashi-ku, Fukuoka 812-8581, Japan; nakayama@agr.kyushu-u.ac.jp

**Keywords:** gut microbiota, SCFA, moderate malnutrition

## Abstract

Malnutrition has been associated with the gut microbiota composition and the gastrointestinal environment. This study aimed to evaluate whether there is a difference in the gut microbiota profile between the normal and undernutrition (considered moderate malnutrition) children and evaluate the gastrointestinal environment observed from the short-chain fatty acid (SCFA) profile. Ten days’ observations were done between normal (*n*:13) and undernutrition (*n*:15) children. The subject’s diet was recorded using a food record. Analysis of the gut microbiota was performed using 16S rRNA gene sequencing targeting the V3-V4 variables region, while the SCFA profile was analyzed using gas chromatography. The result shows that the undernutrition group’s energy intake was lower than in the normal group. Although there was no difference in diversity index and overall gut composition, overexpression of the genera *Methanobrevibacter*, *Anaerococcus*, *Eubacterium*, and *Succinivibrio* was observed in the undernutrition group. Meanwhile, in the normal group, *Ruminococcus* and *Fusobacterium* were found. In both groups, there was also the dominant of *Prevotella* enterotype. Gastrointestinal conditions in the normal group tended to be more acidic compared to the undernutrition group. It occurs due to the high concentration of propionate and butyric acids.

## 1. Introduction

Malnutrition is a condition of imbalance in nutritional intake consisting of undernutrition and overnutrition. Based on the anthropometry, undernutrition consists of stunting (low height-for-age < −2 standard deviation (SD)), wasting (low weight-for-age < −2 SD), and underweight (low weight-for-height < −2 SD). The malnutrition types above can be grouped into moderate (between −2 and −3 SD) and severe (<−3 SD) malnutrition [1]. World Health Organization (WHO) estimates that 160 million children are stunted, and 50 million children are wasting [2]. Several factors can cause malnutrition, ranging from low birth weight, feeding problems, diarrhea, and social factors such as low maternal socioeconomic status. In Indonesia, citing the data from 2018, the number of children who suffered wasting reached 17.7%, with 13.8% for moderate cases and 3.9% for severe ones [3]. It was also found that the number of children who suffered wasting in Yogyakarta is 7.94% [4]. The consequence is reasonably undesirable. Not only do children with undernutrition tend to have a high risk of experiencing cognitive and motor development inhibition, but they also are predicted to have a more vulnerable social life. Undernutrition children are likely to live in poverty and have a low quality of human resources [5,6]. A strategy is needed to prevent cases of undernutrition.

On the other hand, it is estimated that there are more than 10^14^ microorganisms or ten times more than human cells in the human digestive tract. This collection of microorganisms is known as the gut microbiota. In the last decades, research related to gut microbiota has rapidly developed, especially related to its relationship with the host physiology. Gut microbiota in the digestive tract plays a role in helping nutrient metabolism processes, strengthening gut integrity, increasing immune response, and protecting against pathogens [7]. Interestingly, the gut environment influenced by diet, lifestyle, diseases, and medication plays a more vital role in the diversity of gut microbiota composition than its host genetic [8]. Several studies have indicated a relationship between diet and gut composition [9,10,11]. *Bacteroides* enterotypes were found in high animal fat, protein, and saturated fat diet, whereas in diets high in carbohydrates and simple sugar, more *Prevotella* enterotypes were found [12]. Those are closely related in helping host energy metabolism by synthesizing SCFA from the fermentation of oligo and polysaccharides by commensal bacteria [13].

Three hypotheses regarding the correlation of gut microbiota and undernutrition are mentioned by Gordon et al. [14], namely, (1) undernutrition affects the metabolic function as expressed by the composition of the gut microbiota; (2) the innate or adaptive immune system is modulated by gut microbiota activity; and (3) undernutrition is caused by an association between these two factors with other factors, such as availability and abnormalities of nutrient absorption and the presence of enteropathogens. These hypotheses are supported by findings that explain an association of gut microbiota with the incidence of undernutrition [12,15,16,17,18]. However, there was a reference limitation in the gut microbiota composition of the moderate undernutrition children. This gap needs to be filled as an initial stage of identifying the gut microbiota’s role in the incidence of undernutrition. Therefore, this research aimed to determine the gut microbiota and SCFA profile in normal and undernutrition children considered moderate malnutrition. It is expected that the result can prevent changes in status from moderate to severe malnutrition.

## 2. Materials and Methods

### 2.1. Ethical Approval

This study was approved by the Medical and Health Research Ethics Committee, Faculty of Medicine, Public Health and Nursing, Universitas Gadjah Mada (Approval date: 06 November 2019; Protocol number: KE/FK/1303/EC/2019). This research was a ten-day observational study involving normal and moderate undernutrition children under five years old, living in Tirtoadi village, Sleman, Yogyakarta, Indonesia. Informed consent and assent were signed and obtained from the parents or guardians before the study.

### 2.2. Study Population and Design

One hundred children aged under five years old were socialized with the study background and protocol. Participants who signed and submitted assent and informed consent were further screened for the inclusion criteria. Eligible criteria were z-score cut off between −3 and −2 for moderately malnourished children and between −2 and 2 for normal-weight children. The participants should also not consume antibiotics and laxatives a month before the study. Demographic data, including age, sex, and family background, were obtained from the questionnaire, whereas anthropometric measurement was performed for nutritional status. Thirty children were eligible to join the study, but only 28 children finished the study. The eligible participants were observed for ten days and asked to fill out the food record. At the end of observation, stool samples were collected for gut microbiota and SCFA analysis.

### 2.3. Energy and Macronutrient Analysis

Energy and macronutrient intake were analyzed using Nutrisurvey 2007 program (http://www.nutrisurvey.de/). The type and amount of food were inputted on the program and calculated for its daily energy sufficiency. The results were then compared to Indonesian Recommended Dietary Allowance (RDA).

### 2.4. Stool Sample Collection and DNA Extraction

The fresh stool sample was collected at ten days of observation (±1 days) and put into a sterile container tube with 2 mL of RNA-later (Sigma-Aldrich; R0901; Saint Louis, MO, USA). The collected stool sample was then stored at freeze temperature (−40 °C) immediately within 5 h until the analysis day.

DNA extraction was done by a bead-beating method described by Nakayama et al. [19], with modification. Briefly, after RNA-later 10-fold dilution and washing with PBS, the stool sample was mixed with 300 μL of Tris-SDS solution and 500 μL of TE buffer-saturated phenol and subjected to a bead beater (Bead Mill Homogenizer, Benchmark Scientific, China) at a speed of 4000 rpm for 60 s. The obtained supernatant was added with 400 μL of phenol/chloroform/isoamyl alcohol (25:24:1; *v*/*v*) (Sigma-Aldrich; P2069; Saint Louis, MO, USA) and vigorously mix with bead beater 4000 rpm for 90 s, followed with centrifugation at 13,000 rpm for 5 min at 4 °C. After centrifugation, 250 μL of supernatant was mixed with 25 μL of 3 M sodium acetate (pH 5.2) (Sigma-Aldrich; 567422; Saint Louis, MO, USA) and was incubated for 30 min on ice. Three hundred microliter of isopropanol was added and centrifuged at 13,000 rpm for 5 min at 4 °C. The pellet of DNA was washed with 500 μL of ice-cold 70% ethanol and centrifuged at 13,000 rpm for 5 min at 4 °C. The obtained DNA pellet was air-dried prior to suspended in 20 μL of TE buffer (pH 8.0) and stored at –30 °C until use.

### 2.5. 16S rRNA Sequencing

16S rRNA sequencing analysis was done in the Department of Bioscience and Biotechnology, Faculty of Agriculture, Kyushu University, and referred to Nakayama’s protocol [20]. The stool genomic DNA was amplified using TaKaRa ExTaq HS (Takara Bio, Shiga, Japan), targeting the V3-V4 region (F (Bakt_341F): CGCTCTTCCGATCTCTGCCTACGGGNGGGWGCAG, R (Bakt_805R): TGCTCTTCCGATCTGACGACTACHVGGGTATCTAATCC). The obtained amplicons were then used as a template for secondary PCR, which amplification was with barcode-tag primers. Furthermore, the secondary PCR product of 28 samples was mixed and subjected to paired-end sequencing using an Illumina MiSeq v3 chemistry (Illumina inc, San Diego, CA, USA).

### 2.6. Sequencing Data Processing

The Usearch (v.9.2.64) was used to construct operational taxonomic units (OTUs) with 97% identity and to remove PCR chimeras [21,22]. The raw sequence pairs were first merged using fastq_mergepairs script to pass 70–90% of reads. High-quality sequences from the merged sequences were picked using the fasq_filter script with an expected error was set lower than 2.0. Dereplication was performed to find unique sequences using the derep_fulllength script. Furthermore, to clustering OTU and removing chimera, cluster_otus uniques and uchime_ref otus script were used. The taxonomy of each OTU was analyzed using the SINTAX command with Greengene (v.13.5) database (gg_16s_13.5.fa.gz) and a cut-off value of 0.8 [23]. The usearch_gobal script was used to assign OTUs. The downstream analysis was further used with Quantitative Insights into Microbial Ecology (QIIME) virtual-box pipeline software (v.1.9.1). In QIIME software, command summarize_taxa_througy_plots.py and alpha_rarefaction.py were used to assign taxonomy composition for each sample and calculate the alpha diversity index, respectively. We also performed Linear Discriminant Analysis Effect Size (LEfSe) using an online version of Galaxy to visualized specific microbial that were significantly overexpressed in each group as a biomarker [24]. The Linear Discriminant Analysis (LDA) was performed using one-against-all criteria. The LDA score threshold was 2, and the alpha value was 0.1 for Kruskal–Wallis and pairwise Wilcoxon, respectively.

### 2.7. Stool pH and SCFA Analysis

The stool sample was weighed 0.2 g into a 2 mL micro-tube and added with sterile aquabidest water for injection. The stool suspension was then sonicated for 20 min, followed by centrifugation (14,000 rpm, 4 °C, 10 min). The natan was discarded while the supernatant was centrifuge for the second time (14,000 rpm, 4 °C, 10 min). The final supernatant was injected into GC (Shimadzu, GC-2010 Plus) equipped with an FID detector and capillary column (Crossbond polyethylene glycol, 30 m × 0.25 mm × 0.25 µm). The sample injection temperature and detection temperature was 250 °C, with Nitrogen as a gas carrier (flow rate: 38.7 mL/min and pressure: 100 kPa). Stool pH was analyzed using a pH meter (pH Spear Eutech). After calibration, the probe was directly dipped into the stool sample and wait until a stable measured value.

### 2.8. Statistical Analysis

Statistical analysis was performed in R programme (v.4.0.3) and R studio (v. 1.3.1093) using the ggplot2 (v. 3.3.2), corrplot (v. 0.84) and vegan (v.2.5-6) packages. A comparison between the group was analyzed using the Wilcoxon rank-sum test. Spearman correlation was analyzed for selected parameters and visualized as corrplot graph from the corrplot package. Permutational multivariate analysis of variance (PERMANOVA) was used based on Bray–Curtis dissimilarity using the Adonis function with 999 permutations from the vegan package to evaluate the overall gut microbiota composition at OTU and genus level. Betadisper was used to analyze the variance homogeneity between groups before performing PERMANOVA. Non-Metric Multi-Dimensional Scaling (NMDS) based on Bray–Curtis dissimilarity was also used to visualize the difference between groups.

## 3. Results

### 3.1. Participant Characteristics

Twenty-eight participants completed the study and were divided into normal (*n* = 13) group and undernutrition (*n* = 15) group. The normal group consists of 61.54% male and 38.46% female participants; meanwhile, the undernutrition group consists of 46.66% male and 53.33% female participants. Table 1 informs that a significant difference was observed in weight and height between the two groups.

### 3.2. The Difference in Dietary Intake between Normal and Undernutrition Children

**Figure 1:**
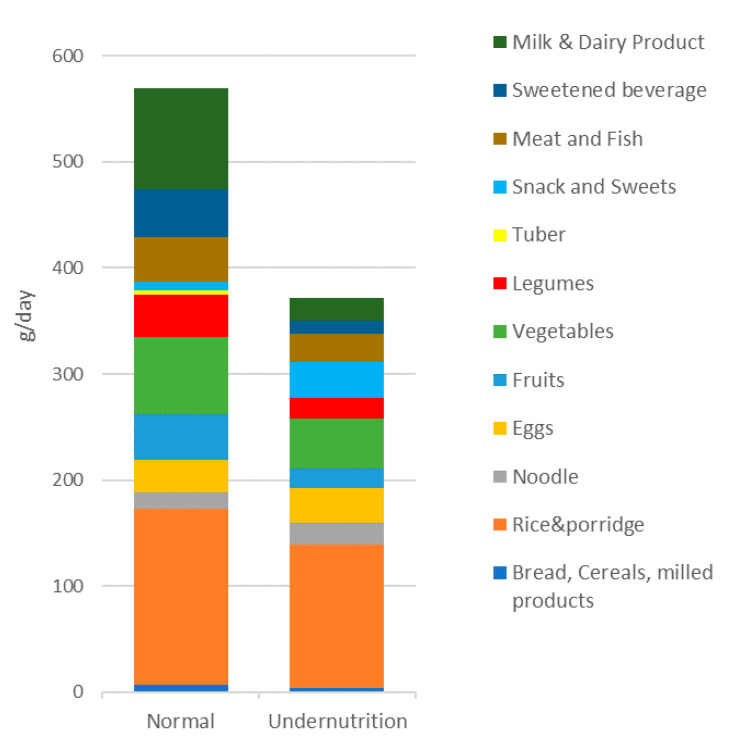
The amount of food consumed per day.

Table 2 shows the daily dietary intake between the two groups, which evaluates ten days of food records. An expressive difference between the two groups in daily dietary intake was observed in all analyzed parameters. The normal group’s energy intake reached 69.18% of Indonesia’s RDA; meanwhile, the undernutrition group only 50.69%. According to Hayati et al. [25], energy intake is sufficient to meet 70% of the RDA. Although the normal group’s energy intake was less than 70%, it was higher than the undernutrition group. Moreover, in both groups, carbohydrates and dietary fiber intake were less than 70% of RDA. Figure 1 shows the subject’s amount of food per day summarized from the shopping list from the Nutrisurvey program. The normal group consumed 569.69 g food/day, higher than the undernutrition group, which only 372.15 g food/day. Interestingly, the undernutrition group tended to consume more snacks and sweets (33.8 g/day). In addition, undernutrition groups consumed fewer vegetables and fruits than normal groups, which is why dietary fiber intake in the undernutrition group was low.

### 3.3. The Difference in Gut Microbiota Composition between Normal and Undernutrition Children

**Figure 2:**
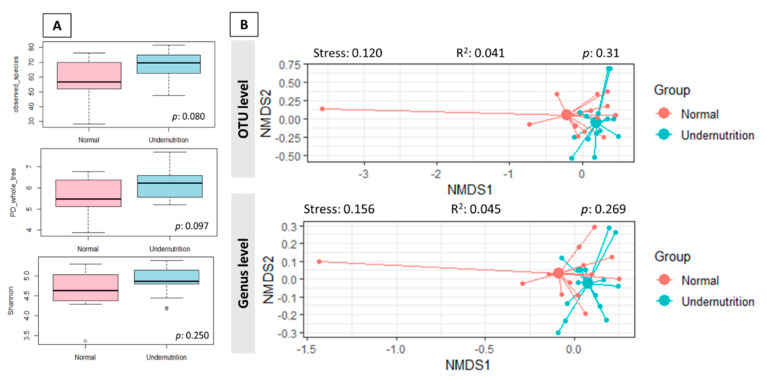
(**A**). Alpha diversity of gut microbiota between normal and undernutrition group. The difference between the group was analyzed using the Wilcoxon rank-sum test (**B**). The Bray–Curtis dissimilarity-based NMDS between normal and undernutrition group. Each data point in the graph stands for a sample. The distance between data points reflects the similarities between samples. The stress factor < 0.2 indicates the reliability of the result.

The gut microbiota between normal and undernutrition was defined by sequencing the V3-V4 region’s 16S rRNA gene. A total read number of 612,159 was obtained from 28 samples, with 21,862.821 ± 4928.863 reads per sample (min–max: 13,951–33,229) resulting 361 OTUs. In total, 11 phyla, 40 families, and 72 genera were detected. Moreover, there was no significant difference in the alpha diversity index (Figure 2A). In addition, overall gut microbiota composition at OTU (PERMANOVA; R^2^: 0.041; *p*: 0.31) and genus (PERMANOVA; R^2^: 0.045; *p*: 0.269) level between groups were not a significant difference. NMDS plot of the gut microbiota profile indicates the tendency of nutritional status grouping the subjects (Figure 2B). The undernutrition samples were grouped event though no clear separation from the normal group.

**Figure 3:**
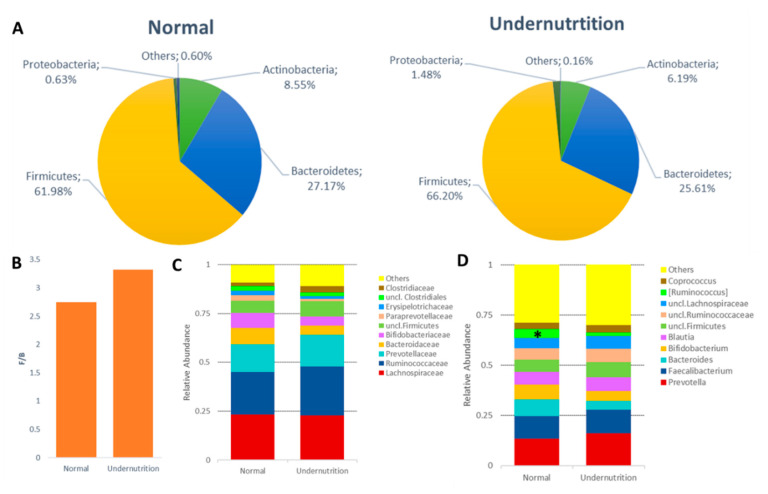
Gut microbiota profile between normal and undernutrition group. (**A**) The difference in the phylum level of gut microbiota between normal and undernutrition groups. (**B**) Firmicutes and Bacteroidetes ratio (F/B) between normal and undernutrition group. (**C**) top 10 relative abundance of family between the group. (**D**) top 10 relative abundance of genus between the group. * means significantly different (*p* < 0.1).

Figure 3A shows the difference of gut microbiota on the dominant phylum level between normal and undernutrition groups. The mean relative abundance of Actinobacteria and Bacteroidetes in the normal group was 8.55 ± 10.36% and 27.17 ± 9.31%, respectively. Whereas in the undernutrition group, both phyla were lower, which was 6.19 ± 4.28% and 25.61 ± 10.98%, respectively. On the other hand, Proteobacteria was more abundant in the undernutrition group (1.48 ± 2.34%) than normal (0.63 ± 0.77%). As the most dominant phylum in the human gut, the proportion of Firmicutes in normal and undernutrition groups was 61.98 ± 15.43% and 66.20 ± 12.82%. The relative abundance of Proteobacteria in the undernutrition group was 2.34 times higher than their normal counterparts. Moreover, Actinobacteria was 1.38 times higher in the normal than undernutrition group. Even though no significant difference was seen (*p*: 0.461), a higher F/B ratio in the undernutrition group (3.32 ± 2.03) over the normal group (2.75 ± 1.65) was observed (Figure 3B).

Apart from the dominant phyla, non-dominant phyla were also identified, which were Cyanobacteria, Elusimicrobia, Fusobacteria, Lentisphaerae, Synergistetes, and Verrucomicrobia. Only one phylum was detected belonging to the Archaea domain, Euryarchaeota, and was identified only in the undernutrition group (3/15). Further taxonomy identification, the bacterial family was overexpressed by Lachnospiraceae, Ruminococcaceae, Prevotellaceae, Bacteroidaceae, and Bifidobacteriaceae (Figure 3C). Family Prevotellaceae was high in both groups over Bacteroidaceae and Bifidobacteriaceae. However, Bacteroidaceae and Bifidobacteriaceae in the normal group tend to have a higher proportion. Furthermore, the most observed bacterial genus in both groups were *Prevotella*, *Faecalibacterium*, *Bacteroides*, *Bifidobacterium,* and *Blautia*. A significant difference was observed (*p*: 0.065) for Ruminococcus belonging to the Lachnospiraceae family, higher in the normal group (Figure 3D).

In addition, the core microbiota genera identified in all groups consist of *Coprococcus*, *Ruminococcus* belonging to the Ruminococcaceae family, *Catenibacterium*, *Dorea*, *Roseburia*, *Clostridium*, *Anaerostipes*, *Lachnospira*, *Oscillospira*, *Eubacterium* (Firmicutes phylum), *Parabacteroides*, *Odoriobacter Paraprevotella* (Bacteroidetes phylum), *Collinsella* (Actinobacteria phylum), *Succinivibrio*, and *Trabulsiella* (Proteobacteria phylum). The overexpression of *Prevotella* over *Bacteroides* and *Bifidobacterium* in both groups was also observed. However, *Bacteroides* and Bifidobacterium were less abundant in the undernutrition group. Interestingly, it was identified that genus *Akkermansia* belonging to Verrucomicrobia proportion was higher in the normal group (9/13), 4.02 times of undernutrition group.

**Figure 4:**
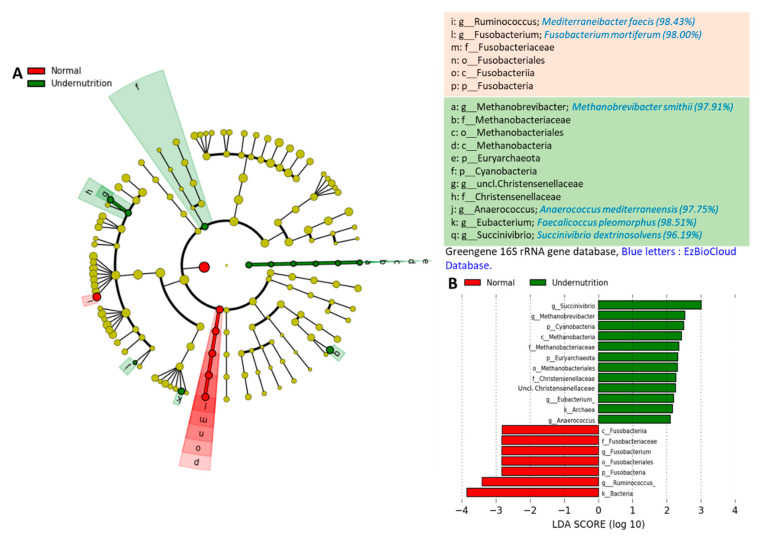
LEfSe showing the difference in gut microbial taxa between the normal and undernutrition group. (**A**) The cladogram shows the difference in gut microbiota between-group; the alphabet indicates the significantly different microbes in each group. Red color refers to the bacteria overexpressed in the normal group, and green color refers to the bacteria overexpressed in the undernutrition group. (**B**) Histogram of LDA score of gut microbiota between-group. Bar length represents the effect size, which explains the differentiating phenotypes between groups. k__, domain; p__, phylum; c__, class; o__, order; f__, family; g__, genus.

Gut microbial profiling to identified bacterial biomarkers between normal and undernutrition groups was performed using LEfSe. A notable difference has been observed in gut microbial between groups, as shown in cladogram and LDA scores (Figure 4A,B). The normal group was dominated by the Fusobacteria phylum (*p*: 0.030), whereas only one genus was identified. The sequence was pinned out by tracking the OTU’s number and compare to EzBioCloud Database, which was identified as *Fusobacterium mortiferum*. Furthermore, the normal group was also characterized by the dominance of genus *Ruminococcus* (*p*: 0.062), which was then identified as *Mediterranibacter faecis*.

On the contrary, the undernutrition group was dominated by Cyanobacteria (*p*: 0.085) and Euryarchaeota (*p*: 0.094) phylum. At the genus level, it was dominated by *Methanobrevibacter* (*p*: 0.094) (Euryarchaeota phylum), *Anaerococcus* (*p*: 0.094), *Eubacterium* (*p*: 0.040) (Firmicutes phylum), and *Succinivibrio* (*p*: 0.089) (Proteobacteria phylum). Those are further identified as *Methanobrevibacter smithii*, *Anaerococcus mediterraneensis*, *Faecalicoccus pleomorphus,* and *Succinivibrio dextrinosolvens*, respectively.

### 3.4. The Difference in SCFA Profile between Normal and Undernutrition Children

**Figure 5:**
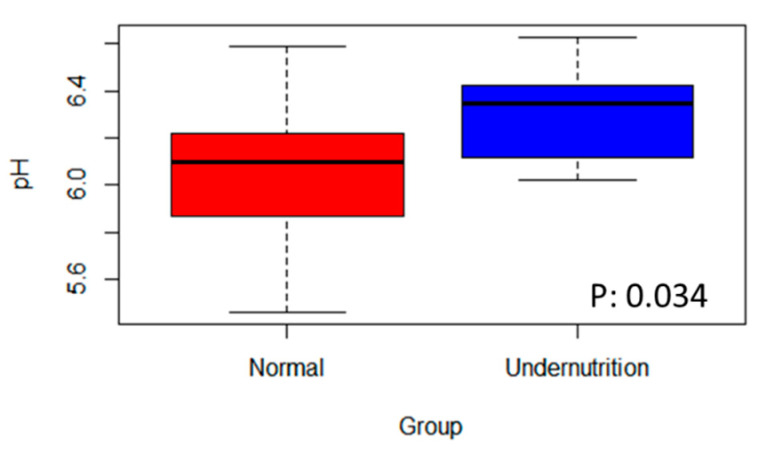
The difference in stool’s pH between normal and undernutrition group.

The SCFA profile was analyzed to evaluate the gut environment between normal and undernutrition groups. Here, the total organic acid was a sum of acetic, propionic, iso-butyric, butyric, iso-valeric, valeric, and iso-caproic acid. Acetic, propionic, and butyric acid was the high concentration SCFA detected from stool samples. The most abundant was acetic acid. A notable difference was observed between-groups (Table 3), propionic and butyric acid, with a high concentration in the normal group. Other than that, stool pH was analyzed to reflect the gut environment acidity due to microbial metabolites. Figure 5 shows the significant difference in stool pH between the group. The normal group had lower stool pH, which was 6.08 ± 0.32, while the undernutrition group was 6.30 ± 0.21.

## 4. Discussion

Based on the weight and height parameters, the undernutrition group can be classified as wasting and stunting with moderate malnutrition severity. This level is marked by a cut-off Z-score between −3 and −2. One of the factors causing undernutrition in children is low energy intake. In this study, the undernutrition category had an energy sufficiency of no more than 70% of the RDA, as well as for carbohydrate and fiber intake in both groups. The low intake of fiber was due to the low intake of vegetables and fruits.

Based on 16S rRNA sequencing results, no significant differences were found regarding the diversity and overall gut microbiota composition between groups. Undernutrition children have low gut microbiota diversity with a higher relative abundance of Proteobacteria [26]. The same result was observed in this study, although the differences found are not significant. The undernutrition group tends to have a higher and lower number of Proteobacteria for Actinobacteria and Bacteroidetes. The results are in line with Monira et al. [15], who found that the relative abundance of Proteobacteria in undernutrition children in Bangladesh was higher than in the normal group, where the dominant genera were *Klebsiella* and *Escherichia*, although it was detected that Bacteroidetes were more dominant in undernutrition children in a study by Gupta et al. [27]. In contrast, Proteobacteria was found to be higher in obese people [28]. Moreover, in line with Méndez-Salazar et al. [28], the F/B ratio in the undernutrition group is higher. A Higher F/B ratio is associated with the incidence of obesity [29,30,31]. The abundance of Firmicutes affects lipid absorption by increasing the number of lipid droplets [29]. It also suggests that Firmicutes modulate calorie absorption’s effectivity by increasing energy harvest capacity [32]. Even though this finding remains unclear, a diet high in sugar and low in fiber in the undernutrition group may cause a high F/B ratio (Table 2 and Figure 1), as stated in research by Méndez-Salazar et al. [28]. In addition, the lower relative abundance of Bacteroidetes may cause the N-glycan pathway deficiency, contributing to the energy extraction efficiency of non-digestible polysaccharides [33,34].

**Figure 6:**
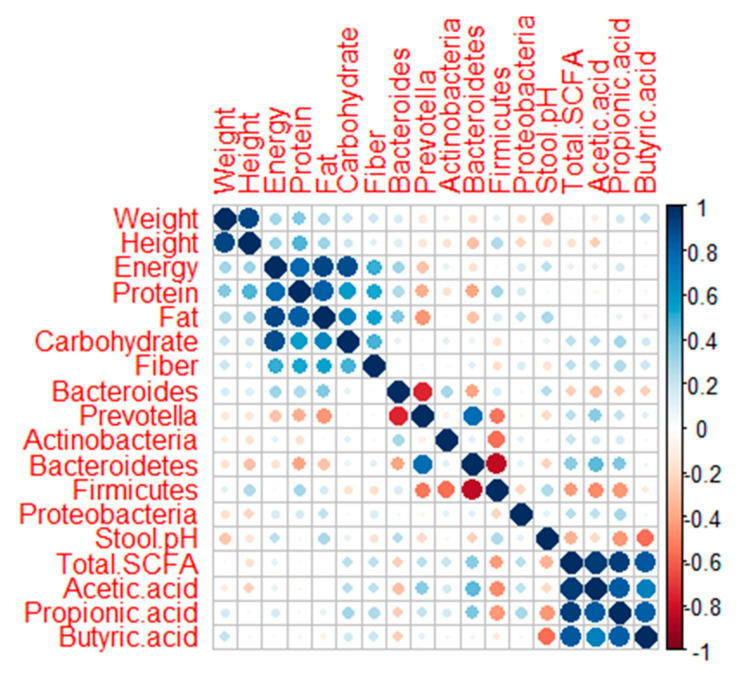
Corrplot showing the correlation between two parameters. The correlation was analyzed using the Spearman method. A bigger circle shows a higher correlation coefficient., A blue circle means positively correlated, while a red circle means negatively correlated.

The dominance of Prevotelaceae, especially the genus *Prevotella,* indicated that both groups had *Prevetolla* enterotypes. These findings are in accordance with Nakayama et al. [19], where children who live in Southeast Asia tend to have the *Prevotella* type, which has a high carbohydrate and fiber diet. Based on the Spearman correlation analysis (Figure 6), *Prevotella* had a negative correlation with the intake of fat (*p*: 0.017; rho: −0.449) and protein (*p*: 0.059; rho: −0.362). On the other hand, *Bacteroides* were positively correlated with fat intake (*p*: 0.051: rho: 0.372) (Figure 6). This result is supported by Khine et al. [35], which explained the change in *Bacteroides* and *Bifidobacterium*’s dominance to *Prevotella* in Indonesian children after weaning. In addition, *Prevotella* type can be found in the younger and elderly groups in Indonesia [36]. In normal groups, they tend to maintain the dominance of *Bacteroides* and *Bifidobacterium*. *Bifidobacterium* is able to protect the gastrointestinal environment and is relatively common in individuals who have normal body weight [37,38,39]. In addition to having a glycan-degrading enzyme and a role in SCFA production, *Bacteroides* also has the ability to degrade animal-derived glycoproteins. This explains why the digestive tract in a diet high in fat and animal protein is dominated by *Bacteroides* [40]. *Bacteroides,* together with the genus *Clostridium*, are able to convert primary bile acid to secondary bile acid [41]. Secondary bile acid can activate TGR5 receptors that play a role in body weight, glucose metabolism, immune system modulation, and liver function [42].

Phylum Bacteroidetes has a significant correlation to propionate concentration [43], as was found in this study. Bacteroidetes had a correlation with acetate (*p*: 0.017; rho: 0.447) and propionate (*p*: 0.042; rho: 0.387) (Figure 6). This study also detected a correlation between the acidic conditions of the digestive tract with the concentration of SCFA, especially butyrate (*p*: 0.002; rho: −0.551) and propionate (*p*: 0.019; rho: −0.439) (Figure 6). The same finding was mentioned by Li et al. [41]. An opposing result was found in research by Kvissber et al. [44], who detected low stool’s pH in severe undernutrition children with carbohydrate malabsorption. The low abundance of good bacteria produces less SCFA, especially butyric acid, resulting in an increment of the colon’s pH and promoting pathogenic growth [45,46]. Pathogenic bacteria can produce lactic and succinate acid, which causes a more acidic digestive environment and damage to the intestinal epithelial [47].

In the digestive tract, SCFAs have the ability to act as an anti-inflammatory, create a selective condition against pathogens, and play a role in homeostasis and energy metabolism [48,49,50]. Butyrate plays a role in the differentiation of intestinal cells, stimulates mucin production, and strengthens the integrity of colonic epithelial cells [41,48], while propionate acts more as an anti-inflammatory [41]. Undernutrition children have weak gut barrier protection and are prone to inflammation and infection [26]. This explains the finding that, in the normal category, they have higher concentrations of propionate and butyrate. This result is supported by Monira et al. [51], who found that children with severe malnutrition had lower SCFA concentrations.

The domination of obligate anaerobe bacteria in the colon provides benefit to the host by producing SCFA. In contrast, the presence of facultative anaerobe bacteria characterizes dysbiosis in the undernutrition group. It is a cyclic correlation, in which a low concentration of SCFA means low selectivity for pathogenic bacteria that can cause epithelial inflammation and affect the nutrition adsorption. The low concentration of SCFA, especially butyric acid, causing a metabolic reorientation of colonocytes toward anaerobic glycolysis and release lactate as well as NO_3_^−^. Thus metabolite caused the epithelial’s inflammation and turned the colon to be more aerobic, thereby increasing facultative anaerobic bacteria [52].

The relative abundance of *M. smithii*, *A. mediterraneensis*, *F. pleomorphus*, and *S. dextrinisolvens* was observed to be dominant in the undernutrition group. The presence of *M. smithii* is associated with the incidence of malnutrition, which is a methanogen bacterium [53,54,55]. Meanwhile, *A. mediterraneensis* is a new species of the genus *Anaerococcus* isolated from an infected vagina [56]. The genus *Anaerococcus* is also found in the skin and nasal cavities and is often involved in infection cases [57]. However, no references could be found regarding the presence of *F. pleomorphus* in the human gut. The genus *Faecalicoccus* has phylogenetic similarities to *Eubacterium cylindroides* (94.4% 16S rRNA sequence similarity with strain LMG 27428T) and reclassification of *Streptococcus pleomorphus* [58]. On the other hand, *S. dextrinisolvens* belonging to the Proteobacteria phylum were identified in the incidence of Bacteremia [59] and known to be dominant in African native gut [60].

*M. faecis* and *F. mortiferum* were found to be more dominant in the normal group. According to Togo et al. [61], *M. faecis* is a reclassification of *Ruminococcus faecis* isolated from human gut. Although the genus *Fusobacterium* is associated with colon cancer [62,63], *F. mortiferum* actually has the ability to produce a bacteriocin-like substance that can inhibit *Salmonella enteritidis* [64]. On the other hand, *F. mortiferum* has been identified as involved in septicemia cases, although these are rare [65].

Regardless of not significantly different, mucosal degrading bacteria, genus *Akkermansia* was detected to be higher in the normal group. This genus is a biomarker of digestive health status [66]. *Akkermansia* can colonize the mucosal layer and degrade it as a carbon and nitrogen source to produce acetate and propionate [67]. In this way, the *Akkermansia* bacteria will not compete with other bacteria in the lumen and not depend on the nutrients from the host’s food [68]. By colonizing this genus, infection of the mucosal layer by pathogens can be prevented. Therefore, this genus plays a very important role in maintaining the gut barrier, especially when enteral nutrition intake is low, such as long term fasting and malnutrition [66].

## 5. Conclusions

An extensive literature discussed gut microbiota’s role in severe undernutrition, characterized by a high abundance of Proteobacteria and low Bacteroidetes. Although there was no difference in gut diversity and overall gut community found in this study, there was a tendency for the composition of gut microbiota in moderate malnutrition to be similar to severe malnutrition, especially phylum Proteobacteria and Bacteroidetes. These findings indicate that changes in the gut microbiota composition had occurred before the child reached severe malnutrition. This study also detected the overexpression of certain bacteria in the two groups that can be used as moderate malnutrition biomarkers. The balance of the gut microbiota plays an essential role in maintaining the digestive environment’s condition, especially in terms of the metabolites produced, namely SCFA. In this case, SCFA has selective properties against unfavorable bacteria. The results of this study are hoped to contribute to preventing malnutrition in children, especially in Indonesia. However, this research has some limitations that can be improved for the next research, such as the use of an old version of downstream analysis software and statistical power to define gut microbiota relative abundance.

## Figures and Tables

**Table 1:** Participant characteristics of the study.

	Normal (n:13)	Undernutrition (n:15)	*p*
Male	8 (61.54%)	7 (46.66%)	
Female	5 (38.46 %)	8 (53.33%)	
Age (Mo)	41.15 ± 14.34	40.73 ± 11.00	0.931
Weight (kg)	14.80 ± 3.98	10.70 ± 1.54	0.000
Height (cm)	95.94 ± 8.98	87.30 ± 6.49	0.007

Data are presented as mean ± SD. A significant difference between the group was performed with the Wilcoxon rank-sum test (*p* < 0.05).

**Table 2:** Daily dietary intake between the two groups.

	Unit	Normal	Undernutrition	*p*	RDA	%RDA	
						Normal	Undernutrition
Energy	kcal	933.99 ± 137.86	684.31 ± 225.95	0.002	1350	69.18	50.69
Protein	g	38.80 ± 7.65	27.25 ± 7.96	0.000	20	194.00	136.27
Fat	g	37.09 ± 9.85	24.57 ± 9.36	0.002	45	82.43	54.61
Carbohydrate	g	111.74 ± 18.00	88.16 ± 31.37	0.016	215	51.97	41.00
Dietary Fiber	g	5.72 ± 1.93	3.35 ± 1.53	0.010	19	30.12	17.61

Data are presented as mean ± SD. A significant difference between the group was performed with the Wilcoxon rank-sum test (*p* < 0.05).

**Table 3:** Short Chain Fatty Acid Profile between the two groups.

Organic Acid	Mmol/g Feces (Mean ± SD)		*p*
	Normal	Undernutrition	
Total organic acid	33.54 ± 13.86	27.01 ± 16.74	0.112
Acetic acid	18.21 ± 8.14	16.75 ± 11.95	0.279
Propionic acid	7.43±3.61	5.08±3.74	0.025
Butyric acid	5.14±2.46	3.35±1.73	0.033

Data are presented as mean ± SD. A significant difference between the group was performed with the Wilcoxon rank-sum test (*p* < 0.05).

## Data Availability

The data presented in this study are available on request from the corresponding author.
